# Surgical Notes

**Published:** 1860-04

**Authors:** E. Andrews

**Affiliations:** Professor of Surgery in the Medical Department of Lind University


### SURGICAL NOTES.

By E. ANDREWS, M. D.,
Professor of Surgery in the Medical Department of Lind University.

*Fracture of the Patella.*—Mr.——, aged 23 years, fell and struck his knee upon a stone, fracturing the right patella transversely, and of course losing instantly the power of extending the leg. On examination I found the two fragments widely separated, but the patient was absolutely free from all pain and tenderness in the injured part. I drew the fragment together with a figure of eight bandage, and subsequently devised a pair of concave yokes for retaining fractured surfaces in contact. No inflammation whatever followed, and reparative action was very slow. After many weeks there was a feeble ligamentous union, and the patient began to walk about.

One day in making a little stronger muscular effort than usual, he ruptured the ligament, and was immediately reduced again to his former condition. This time I found him suffering considerable pain. On examining the patella I found that there was a marked thickening of the areolar tissue about it, through which hypertrophied tissues the fragments seemed to get more abundant supplies of blood and nervous influence than in the natural state. A considerable inflammation followed, and in a much shorter period than before there was a firm ligamentous union. The treatment was the starch bandage, which served its purpose admirably, and at the same time allowed the patient to get about and do some business.

The study of this case suggests some thoughts respecting the causes which prevent union so universally in transverse fractures of the patella. It is believed by many that these causes are, first, the presence of synovia between the fractured surfaces, and secondly, the separation of the fragments.

Now the synovial fluid does not prevent the union of oblique fractures of long bones when they open into joints, though it is abundantly present; nor is there any difficulty, usually, in keeping the fragments of a patella in contact, if the leg is kept extended. Some other reason, therefore, exists for the want of reparative power. If we examine the anatomical relations of the patella, we shall perceive what this reason is. The deep surface of the bone is entirely occupied by a synovial lining, and therefore receives no blood vessels except such as creep under the membrane at the edges. The external surface is in like manner covered by the great bursa, which equally separates it on that side from direct vascular supplies. The two lateral borders are connected with the capsular ligament of the knee-joint, which is fully furnished with vessels. The lower border gives origin to the dense *ligamentum patellæ*, a tendinous structure very poor in vascular supplies. The upper border alone receives a full amount of nutrition, in consequence of its attachment to the *quadriceps femoris* muscle. From this attachment, especially where the two *vasti* muscles are inserted, the patella obtains nearly the whole of its nutrition. It follows, therefore, that in a transverse fracture, the lower fragment will be almost cut off from its nutrient vessels, and is in fact very much in the same condition as the head of the femur in the intracapsular fracture of the neck of that bone. It has therefore no power to throw out plastic material, and fails to effect a bony union, however faithful may be the surgeon.

This view suggests almost irresistably the propriety of resorting to some operative proceedure in these cases, by which a temporary inflammation and vascularity may be induced in the tissues around the lower fragment, which will supply it with blood during the time of treatment. I think that in this way many fractured patellas might be brought to form a bony union which now fail of it. The irritation might be produced sub-cutaneously by the careful introduction of a small stilet, or other instrument.

The obvious danger of exciting an inflammation in the knee joint is the great obstacle to such an operation, and I know of no experiments as yet which would show how this danger may be avoided, but as the joint itself need not be penetrated, perhaps this operation will ultimately be found feasible.

